# *COL1A1::PDGFB* fusion‐associated uterine fibrosarcoma: A case report and review of the literature

**DOI:** 10.1002/cnr2.1969

**Published:** 2024-01-26

**Authors:** Simone Rota, Andrea Franza, Chiara Fabbroni, Biagio Paolini, Francesca Gabriella Greco, Alessandra Alessi, Barbara Padovano, Paolo Casali, Roberta Sanfilippo

**Affiliations:** ^1^ Department of Medical Oncology Fondazione IRCCS Istituto Nazionale dei Tumori di Milano Milan Italy; ^2^ Department of Interventional Radiology Fondazione Istituto di Ricovero e Cura a Carattere Scientifico (IRCCS) Istituto Nazionale Dei Tumori Milan Italy; ^3^ Department of Nuclear Medicine Fondazione IRCCS Istituto Nazionale Tumori Milan Italy; ^4^ Medical Oncology Università degli Studi Milan Italy

**Keywords:** *COL1A1::PDGFB*, fibrosarcoma, Imatinib, soft tissue sarcomas, translocation t(17;22)(q22;q13), uterus

## Abstract

**Background:**

Mesenchymal neoplasms of the uterus encompass a diverse group of tumors, with varying characteristics and origins, collectively accounting for 8% of uterine malignancies. The most common variants include uterine leiomyosarcoma, low‐grade and high‐grade endometrial stromal sarcoma, adenosarcoma, and undifferentiated sarcoma. Clinical presentation is often nonspecific and can lead to delayed diagnosis. Uterine sarcomas are generally aggressive, resulting in poorer prognosis compared to carcinomas. Recent advances in molecular techniques, such as next‐generation sequencing (NGS), have led to the identification of new subtypes of uterine sarcomas, including *COL1A1::PDGFB* fusion‐associated fibrosarcoma, which has a specific chromosomal translocation t(17;22)(q22;q13). Imatinib, a tyrosine kinase inhibitor (TKI), is an effective treatment for dermatofibrosarcoma protuberans (DFSP), marked by this translocation.

**Case:**

We present the case of a 42‐year‐old woman diagnosed with *COL1A1::PDGFB* fusion‐associated uterine fibrosarcoma. The patient underwent total hysterectomy and excision of the tumor, initially misdiagnosed as a low‐grade leiomyosarcoma. Subsequent histological examination, immunohistochemistry, and fluorescence in situ hybridization (FISH) confirmed the diagnosis. After 10 months, disease recurrence was detected, and Imatinib therapy was initiated at a dose of 400 mg daily. An allergic reaction led to a temporary discontinuation, but upon resumption with appropriate medication, a positive radiological response was observed. The patient achieved a complete remission after 2 years and is still on Imatinib treatment.

**Conclusions:**

*COL1A1::PDGFB* fusion‐associated uterine fibrosarcoma is an extremely rare mesenchymal neoplasm. In a case we present herein, we treated a patient with imatinib as first‐line medical therapy. The patient is currently in complete remission after 37 months from treatment start. To the best of our knowledge, this represents a unique observation. We also provide a detailed literature review of the published cases so far. Prospective case series are needed to further understand the natural history of these tumors and optimize treatment strategies.

## 1 INTRODUCTION

Mesenchymal neoplasms of the uterus comprise a heterogeneous group of tumors, with different morphologies and immunophenotypes, collectively accounting for 8% of uterine malignancies.^1^ In recent years their incidence has increased, mainly due to the new diagnosing techniques and population aging.^1^ The most frequent mesenchymal malignancies originating from the uterus are uterine leiomyosarcoma (commonly originating from the smooth muscle in myometrium), low‐grade and high‐grade endometrial stromal sarcoma (originating from the endometrial stroma), adenosarcoma and undifferentiated sarcoma.^2^, ^3^, ^4^ Leiomyosarcoma is the most common variant and is characterized by an aggressive behavior, with a 5‐year survival rate in the 50% range in available series. Undifferentiated endometrial sarcoma is highly aggressive, with a low 5‐year survival rate. On the other hand, endometrial stromal sarcoma show a more indolent course if they are low‐grade, with a high survival rate, though with a significant risk of relapse.^5^

Clinical presentation of mesenchymal uterine tumors is non‐specific and hystotype‐dependent. Uterine sarcomas often present as a pelvic mass, which may be associated with abdominal pain and/or vaginal bleeding.^6^ Although less frequent than carcinomas, these tumors have a worse prognosis because of their aggressiveness, resulting in higher mortality rates.^7^

In addition to the four most common histotypes, other mesenchymal tumors can be also found in the uterus (i.e., peripheral nerve sheath tumors).^8^ Furthermore, thanks to the novel molecular techniques available in the last few years, namely next generation sequencing (NGS), it has been possible to identify new subtypes of uterine sarcoma.^9^ Among these, the uterine collagen type 1 (*COL1A1*) and platelet‐derived growth factor beta chain (*PDGFB*) fusion‐associated fibrosarcoma gained interest among clinicians and researchers because of its specific chromosomal translocation t(17;22)(q22;q13). This genomic alteration is recurrently found in pediatric giant cell fibroblastoma and dermatofibrosarcoma protuberans (DFSP).^10^, ^11^ The latter is the most common dermal sarcoma, although it accounts for 18% of all cutaneous soft tissue sarcomas.^12^ Most frequently located in the trunk (42%) and with a higher incidence rates in women, it shows indolent behavior with a low level of aggressiveness (except for the fibrosarcomatous form, which progresses from the classical on the basis of transcriptional reprogramming and is characterized by a more aggressive clinical presentation).^12^, ^13^, ^14^ Nonetheless, it is considered to be of intermediate malignancy, because of its significant recurrence rate (25% at 5 years).^15^ While it can arise at any age, it affects mainly adults between the third and the fifth decades.^12^ Metastases, although rare (<1%), may be fatal.^12^

Even if the mainstay of treatment of these ultrarare uterine mesenchymal tumors has not been established yet, the chromosomal translocation t(17;22)(q22;q13) could be a key target for the treatment of *COL1A1::PDGFB* fusion associated uterine fibrosarcoma.^16^, ^17^ Indeed, *COL1A1::PDGFB* fusion, as extensively studied in the DSFP setting, leads to an overproduction of PDGFB aberrant leading downstream to activation of the Ras MAPK and PI3K‐AKT‐mTOR pathway, thus resulting in an uncontrolled cell growth. The tyrosine‐kinase inhibitor (TKI) Imatinib targets PDGFBR effectively.^17^

Imatinib is a well‐known TKI firstly employed in the treatment of chronic myeloid leukemia, as it specifically targets the BCR‐ABL fusion which is pathognomonic in that condition.^18^, ^19^ The drug is also effective in gastrointestinal stromal tumors (GIST), where it radically changed their prognosis and management, becoming the standard therapy both in the perioperative and metastatic setting.^20^, ^21^ This is due to the fact that GIST are marked by an activating mutation of KIT or PDFGRA, both targeted by the drug. Imatinib is usually given at the dose of 400 mg, but various schedules have been investigated. Typical adverse events include edema, frequently periorbital, (74% of cases), nausea (52%), diarrhea (45%), arthromyalgias (40%), fatigue (35%), dermatitis or rash (31%), headache (26%) and abdominal pain (26%). However, Imatinib therapy is often well tolerated by patients.^22^

Hereby, we present the case of a woman diagnosed with *COL1A1::PDGFB* fusion‐associated uterine fibrosarcoma treated with Imatinib at the time of post‐surgical recurrence. With the aim of increasing the knowledge of this ultrarare condition and a complete review of available literature are also provided. To the best of our knowledge and considering the poor data available, this is the first case described in the literature where Imatinib has been used in first‐line treatment of *COL1A1::PDGFB* fusion‐associated uterine fibrosarcoma.^23^, ^24^, ^25^, ^26^, ^27^, ^28^

## 2 CASE PRESENTATION

In October 2019, a 42‐year‐old woman with no previous relevant clinical history was admitted to a surgical unit due to the persistence of abdominal heaviness sensation during the last few months. A CT scan was performed, thus revealing a growing pelvic mass of 16 cm, suspected to be a gynecological malignancy. The patient subsequently underwent laparotomic total hysterectomy, and a radical excision of the neoplasm was performed. The first histologic exam, performed in a non‐reference center, defined the lesion as a low‐grade leiomyosarcoma of the uterus. Shortly thereafter, due to the rarity of the condition, the patient was referred at our cancer center (National Cancer Institute of Milan), where a pathologic review was performed Table 1.

**TABLE 1:** Timeline of patient's clinical history.

Date	Relevant medical Event	Effect or detail
October 2019	Total hysterectomy	Diagnosis of low‐grade leiomyosarcoma
March 2020	Second opinion at our cancer center	Final diagnosis of *COL1A1::PDGFB* fusion associated uterine fibrosarcoma
June 2020	CT scan and PET with fluoro‐desoxyglucose (FDG)	Local recurrence
June 2020	Start of medical treatment	Imatinib 400 mg/die
June 2020	Allergic reaction	Drug interrupted
July 2020	MRI‐abdomen and pelvis and PET fdg	Initial metabolic response
August 2020	Resume of medical treatment	Imatinib 400 mg + steroid and antihistamine coverage
October 2020	MRI‐abdomen and pelvis	First disease response
November 2022	MRI‐abdomen and pelvis	Complete radiological response

The pathological report described a spindle cell neoplasm with mild to moderate cytological atypia, focal myxoid areas, and inflammatory infiltrate consisting of lymphocytes and eosinophilic granulocytes. Mitotic activity varied throughout the tumor (till 5–6 mitosis per 10 high power field).

**FIGURE 1:**
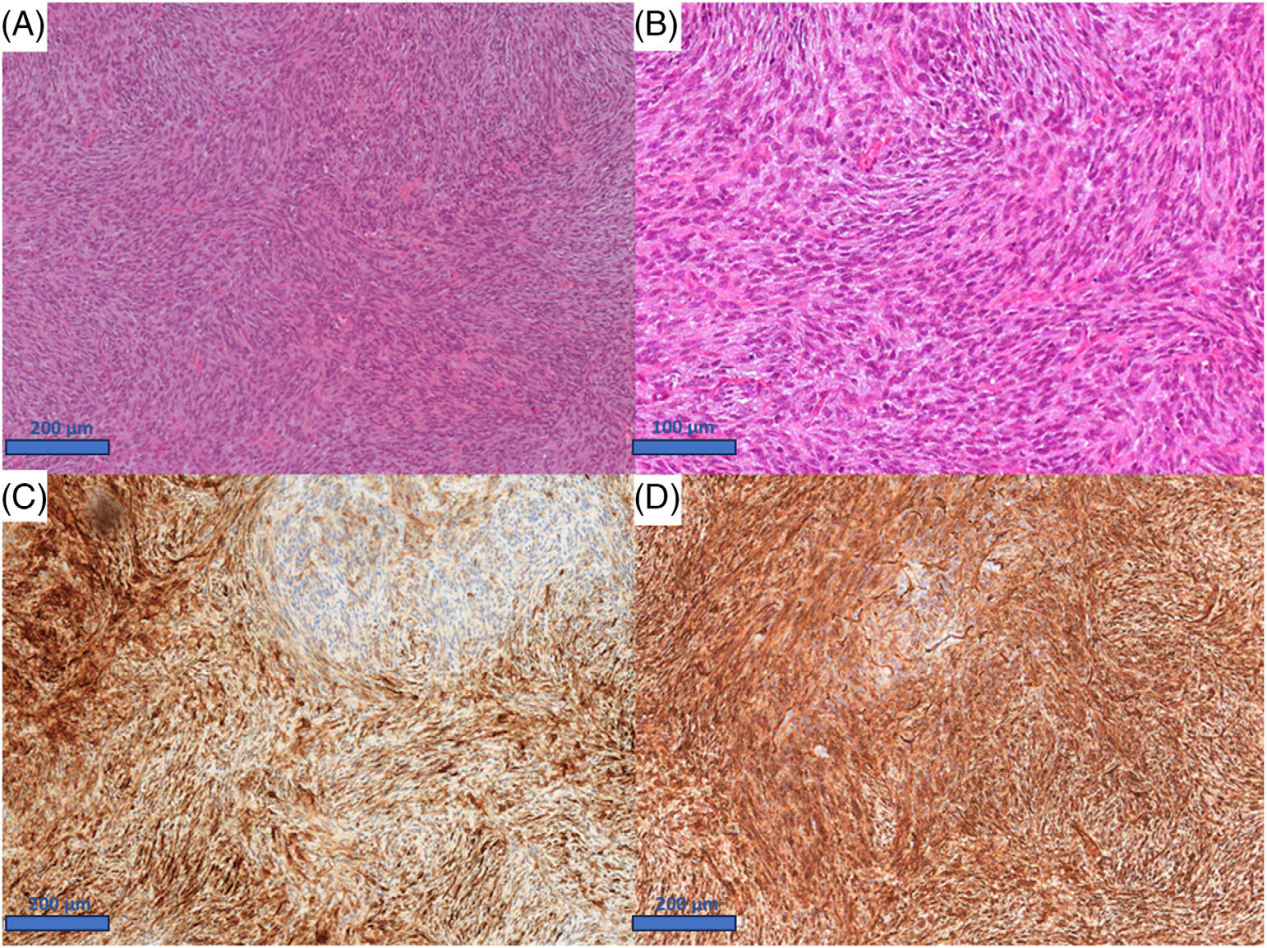
Morphological and immunohistochemical features of the primary tumor. (A) Uterine mesenchymal tumor composed by spindle cells with a storiform to whorled pattern (H&E, 10×). (B) Spindle cells show eosinophilic cytoplasm, monomorphic and ovoid nuclei, very low to absent mitotic activity (H&E., 20×). (C) Patch positivity for CD10 (10×); (D) Strong and diffuse positivity for CD34 (10×).

Strong positivity for CD34, CD10 and p16 was detected on immunohistochemistry, with focal actin positivity and negative staining for desmin, caldesmon, BCL1, STAT‐6, and B‐COR (Figure 1).

**FIGURE 2:**
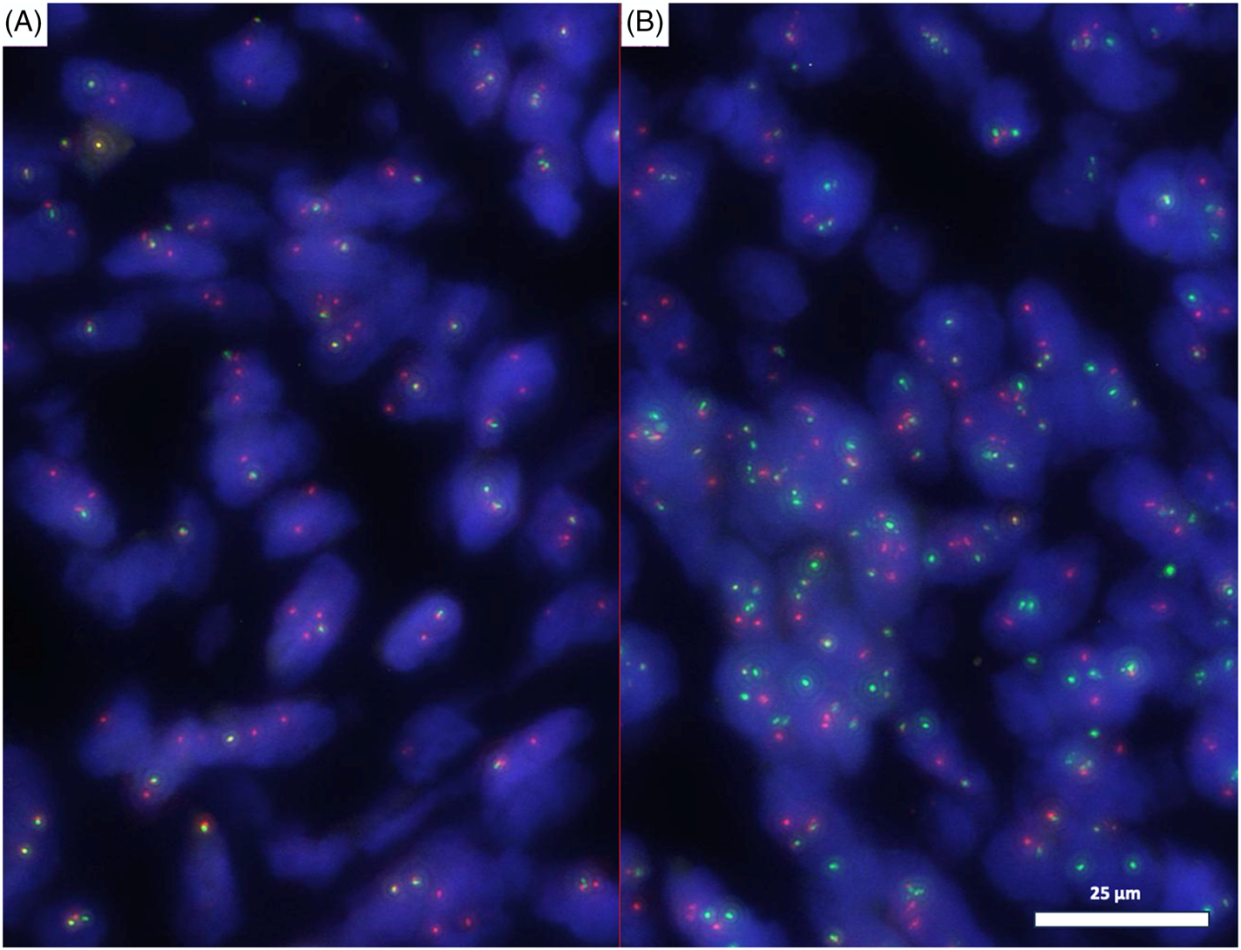
(A) “Break apart *PDGFB* FISH figure”: here it is represented an unbalanced translocation with loss of the 5′ probe part of the PDGFB gene (green one) and gain of probe copies at 3′ (orange). This pattern is typical of DFSP. (1000×). (B) “Dual fusion‐dual color *COL1A1::PDGFB FISH figure*.” Here it is possible to see the fusion: (orange: COL1A1/ green: PDGFB). (1000×).

Such findings were not compatible with a smooth muscle lesion and were unusual for an endometrial stromal lesion, so that a genomic test searching for chromosomal translocations was performed. While fluorescence In Situ Hybridization (FISH)—testing was negative for translocations of JAZF1, BCOR and YWHAE genes, it showed the presence of an unbalanced PDGFB translocation and *COL1A1::PDGFB* fusion (Figure 2). A final diagnosis of *COL1A1::PDGFB* fusion associated uterine fibrosarcoma was thus rendered.

Two months after surgery whole body computed tomography (CT) excluded local recurrence or metastatic disease localization. Given the absence of distance metastases or local recurrence, as well as the low grade of the malignancy, a close follow‐up program was selected.

**FIGURE 3:**
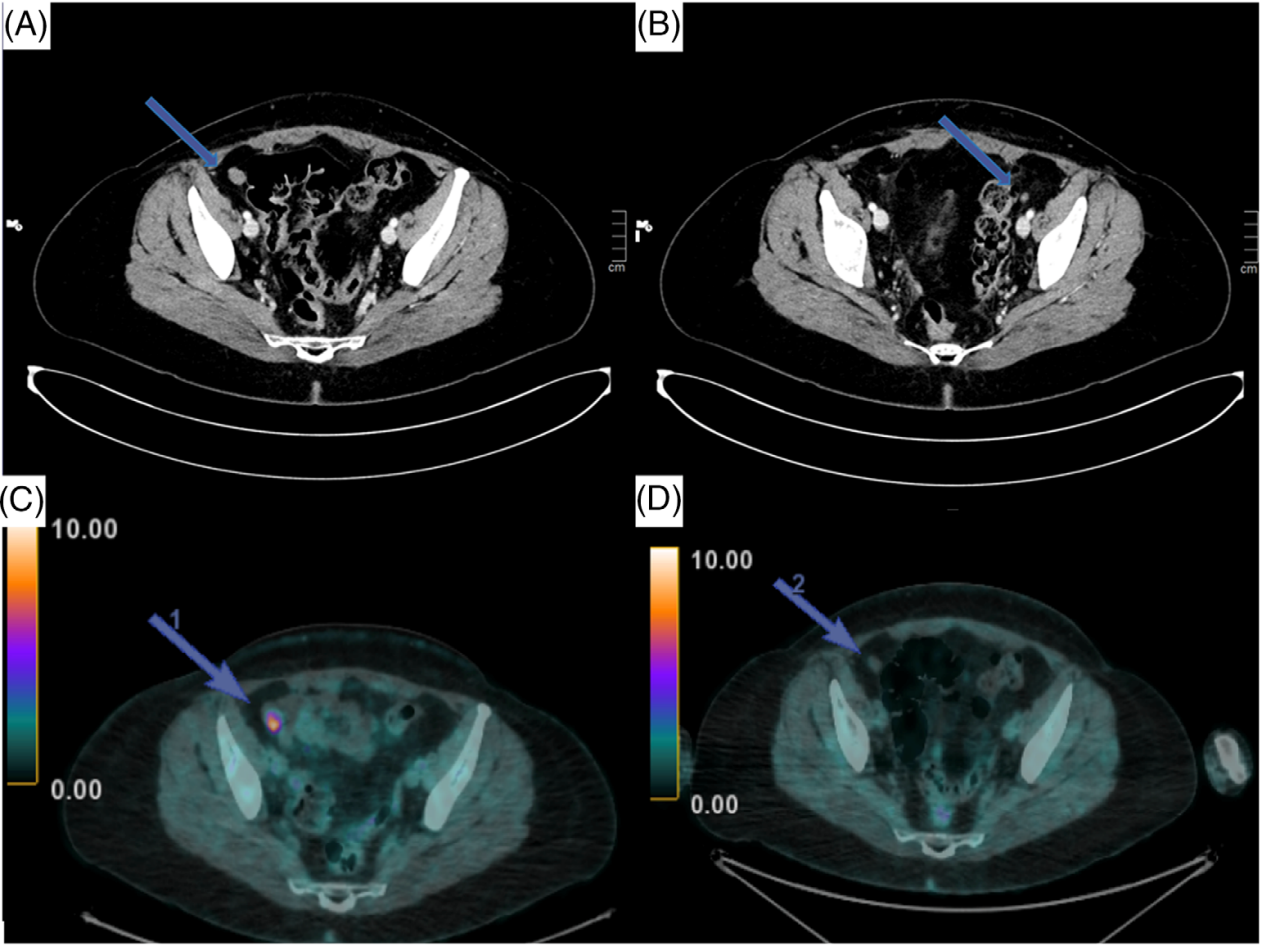
CT scan and FDG PET of the local recurrence. (A) June 2020: CT scan showing local recurrence in the right pelvis; (B) June 2020: CT scan: evidence of local recurrence in the left pelvis; (C) June 2020: PET with FDG showing local recurrence in the right pelvis; (D) July 2020: PET with FDG: evidence of initial response after few days of therapy.

Ten months after surgery, a whole body CT and fluorodeoxyglucose (FDG) positron emission tomography (PET) scans showed disease recurrence to the right pelvic (18 mm) and left emipelvis (7 mm) (Figure 3).

Due to the evidence of recurrent disease, a multidisciplinary discussion was carried out with the gynecologists and abdominal surgeons. Because of the site of disease, radical surgery was considered too demolitive, and a systemic attempt was made. Considering the detection of *COL1A1::PDGFB* translocation, therapy with Imatinib at the dosage of 400 mg‐daily p.o. was started. Unfortunately, due to an allergic reaction (non‐itchy maculopapular erythema diffuse approximately over 30% of the skin surface without other associated symptoms), the drug was interrupted after 10 days. Once anti‐histaminic and steroidal therapy was administered, the patient shortly recovered from the adverse event. We decided to keep TKI suspended until complete resolution of the adverse event.

Thirty days after imatinib discontinuation, a new CT and a PET scan were performed. Unexpectedly, an initial response to treatment was detected (Figure 2). Given the evidence of radiological response and the previous allergic reaction, it was decided, in agreement with gynecologists and abdominal surgeons, to resume Imatinib with an adequate steroidal and antihistaminic coverage (25 mg of Prednisone and 10 mg of Cetirizine).

A close monitoring program with monthly visits was initially set up so that clinical conditions could be monitored due to the prior allergic reaction. Subsequently, four‐monthly program with abdominal magnetic resonance imaging (MRI) was set up.

In October 2020, a new MRI showed a millimetric disease response in both lesion sites, starting a gradual trend of radiological response.

Two years later, a complete radiological response was achieved at MRI.

Treatment with imatinib is still ongoing with optimal tolerance. A decalage of the prednisone dosage was gradually made till the current dosage o 5 mg/daily. Instead, daily 10 mg of cetirizine therapy is still on. No more relevant adverse effects were reported.

## 3 LITERATURE REVIEW

We performed a literature search in the most popular medical literature databases (PubMed®, Embase®, and Google Scholar®), retrieving a total of nine reported cases of *COL1A1::PDGFB* fusion‐associated uterine fibrosarcoma.^23^, ^24^, ^25^, ^26^, ^27^, ^28^

All reported cases diffusely expressed CD34 and were negative for desmin. When tested, S‐100, caldesmon, TRK, ER, and PR were negative as well. In two cases SMA was focally positive^24^ and the same was for P16.^26^ Moreover, CD10 was detected in the current case and described as focally positive in the cases reported by Grindstaff et al. and Panwar et al. and in the one of Chapagain et al.^23^, ^24^, ^28^ Table 2.

**TABLE 2:** Immunohistochemical features.

	CD34	S‐100	ER	PR	desmin	caldesmon	SMA	CD10	TRK	P16
Current Case	Positive (+)	Not available (NA)	NA	NA	Negative (−)	−	Focal positive	+	NA	+
Panwar et al.	+	−	−	−	−	−	−	Focal positive	NA	NA
Grindstaff et al.	+	NA	NA	NA	−	−	Focal positive	Focal positive	NA	−
Croce et al.	+	−	−	−	−	NA	NA	NA	−	NA
	+	−	−	−	−	NA	NA	NA	−	NA
	+	−	−	−	−	NA	NA	NA	−	NA
Linghui Lu et al.	+	−	−	−	−	−	−	−	−	+
Hogeboom et al.	+	−	−	−	−	−	−	−	−	−
Chapagain et al.	+	−	Focal positive	Focal positive	−	−	−	+	NA	NA

Clinical and pathological features of patients described, as well as outcome data, are shown in Table 3. Median age of patients was 50 years (range 43–82) and the most frequent primary site was uterus (5). Three patients had abdominal pain as their first symptom, while three complained for metrorrhagia and one reported mass sensation. No clinical information regarding early symptoms was available in the three cases reported by Croce et al. Most patients (7) were alive at the time of cases publication, while only two were dead. Four out of seven alive patients had no evidence of disease, two were alive with radiologically evident malignancy and in one case this information was not available.

**TABLE 3:** Clinical‐pathological features and outcomes of the case reports published in the literature.

Article	Age	Site	Early symptoms	Nuclear atypia	Mitosis per 10 HPF	Necrosis	Death	Clinical condition	OS (months)	DFI (months)
Current Case	44	Uterus	Abdominal pain and mass sensation	Mild/moderate	From 5 to 6	No	No	Not evidence of the disease (NED)	42	8
Panwar et al.	58	Lower uterus and cervix	Abdominal pain	Moderate	15	Yes	Yes	Dead of the disease (DOD)	13	4
Grindstaff et al.	43	Uterus	Heavy menses	Not available (NA)	Up to 45	Yes	No	Alive without disease (AWD)	NA	NA
Croce et al.	82	Cervix	NA	Mild	8	Yes	No	NED	10	NA
	60	Cervix	NA	Mild/moderate	20	No	Yes	DOD	34	NA
	48	Uterus	NA	Moderate	20	No	No	NA	NA	NA
Linghui Lu et al	57	Cervix	Metrorrhagia	Mild/moderate	30	No	No	NED	6	6
Hogeboom et al.	50	Uterus	Metrorrhagia and abdominal pain	Moderate	54	Yes	No	NED	1	1
Chapagain et al.	43	Uterus	Dysmenorrhea and Metrorrhagia	Mild/moderate	12–22	No	No	Alive with stable disease (ASD)	8	2

Median reported mitotic rate per 10 HPF was 20 (range 5–54) and half of reported cases (4) had necrotic areas.

In two cases^24^, ^25^ overall survival (OS) was not inferable, while in the other five median OS (mOS) was 10.0 months (range 1–42).

Table 4 shows the clinical management in these patients. Three cases reported by Croce et al. did not provide clinical information.

**TABLE 4:** Possible misdiagnosis and clinical management.

Article	Misdiagnosis	Treatment	Type of medical therapies	Imatinib	Time since the start of Imatinib (months)
Current case	Yes	Surgery, tyrosine kinase inhibitor	Imatinib	Yes	32
Panwar et al.	No	Surgery, aromatase inhibitor, chemotherapy	Letrozole, gemcitabine + docetaxel	No	
Grindstaff et al.	Yes	Surgery, chemotherapy, target therapy, TKI	Gemcitabine + Docetaxel, Doxorubicin + Olaratumab, Trabectedin, Pazopanib, Imatinib.	Yes	10
Linghui Lu et al.	No	Surgery	None	No	
Hogeboom et al.	No	Surgery	None	No	
Chapagain et al.	No	Surgery, chemotherapy	Doxorubicin Imatinib	Yes	4

All patients with treatment details underwent surgical resection at the time of diagnosis, and four of them subsequently started systemic medical treatment for recurrence. Among patients with post‐surgical recurrence, Imatinib was never employed as first‐line therapy. In the case reported by Grindstaff et al., instead, it was used as a further therapeutic line with an impressive radiological response.^24^

Of note, in the case reported by Grindstaff et al., the initial diagnosis was uterine leiomyosarcoma, and this led to the initiation of a gemcitabine plus docetaxel regimen.

## 4 DISCUSSION

The case reported herein refers to a patient with a *COL1A1::PDGFB* fusion‐associated uterine fibrosarcoma. This is an extremely rare and recently described mesenchymal neoplasm of uterine origin^12^, ^13^, ^14^, ^15^, ^16^, ^25^ A correct pathological diagnosis complemented by proper molecular assessments was obviously crucial. The finding of the same chromosomal translocation, typical of DFSP, led us to effectively treat this patient with Imatinib.^23^ The patient is currently in complete remission after 37 months from treatment start. This represent an unique observation, as only another case has been reported so far in literature, but with a limited follow‐up.^24^

As proved in our literature review, *COL1A1::PDGFB* fibrosarcoma, in addition to the presence of the *COL1A1::PDGFB* fusion, consistently exhibit a positivity for CD34 and manifest a comparable histological pattern to DFSPs, characterized by a cellular proliferation featuring interwoven fascicles of spindle cells showing mild atypia and increased mitotic activity.^29^ In relation to the strong CD34 positivity, it has been proposed to incorporate it within the routinely employed panel of immunostains for the assessment of uterine sarcomas.^28^ if not routinely, it might be reasonable to test CD34 in the presence of negative desmin and caldesmon, when then the possibility of leiomyosarcoma diagnosis is excluded.

*COL1A1::PDGFB* fusion is responsible for abnormal activation of the PDGFB pathway, and this explains the efficacy of Imatinib in our case.^26^ In DFSP, where the translocation is pathognomonic, a recent review showed that imatinib led to important radiological responses in 60% of advanced, regardless of the daily dose, with good tolerability.^16^ This was the rationale for using Imatinib in our patient.

Namely, we used Imatinib as a first‐line medical therapy in this metastatic patient, given the extent of foreseeable surgery and the limited potential of conventional medical therapies available in sarcomas, generally able to provide response rates lower than with Imatinib in DFSP.

The patient discussed in our paper is still undergoing imatinib therapy after a complete response. Hence, an issue has to do with the continuation of treatment, in the lack of evidence. It may be prudent to contemplate a treatment strategy akin to that of dermatofibrosarcoma protuberans (DFSP). In line with Stacchiotti et al., in DFSP, imatinib may not effectively eliminate metastatic disease, as demonstrated by certain case series where all patients pre‐treated with imatinib subsequently experienced relapse post‐surgery. Additionally, the role of surgery itself remains limited.^29^ Consequently, the decision to persist with imatinib therapy even with a radiological complete remission appears to be a reasonable choice.

Interestingly, the molecular landscape in this tumor was accompanied by a histological appearance close to DFS. This was observed also in the other published cases.^12^, ^13^, ^14^, ^15^, ^16^ Indeed, the tentative label of “*COL1A1::PDGFB* fusion‐associated uterine fibrosarcoma” is currently used for these tumors. Croce et al. categorized it as a new entity due to its different site of origin and the significant mitotic activity as compared to DFSP. This leads to a more aggressive clinical behavior.^12^, ^13^, ^14^, ^15^, ^16^, ^25^ Thus, these tumors seem to imply a relatively specific clinical course, in addition to the peculiar sensitivity to Imatinib. So while the molecular landscape per se may not be the only determinant in defining a medical condition, here the pathologic appearance, the molecular landscape, the peculiar sensitivity to a medical therapy may well define a distinct entity.^30^ On the other hand, it is left to learn which is the complete natural history of these tumors. To this end, probably only prospective case series analyses can be of help.

## 5 CONCLUSIONS

Whatever the classification of this new entity, we know that Imatinib medical therapy can be highly active in the exceedingly rare *COL1A1::PDGFB* fusion‐associated uterine fibrosarcoma. In our case, the molecular assessment was prompted by an atypical histological appearance on pathological review at a sarcoma reference centre and this underlines the need for pathologic awareness about this ultra‐rare entity.

Considering the presented case, as well as the rarity of this diagnosis and the little literature available:

1. High expertise pathologists are required to guarantee a prompt and correct diagnosis of such a rare neoplasm. Rare cancer patients should be referred to high volume reference cancer centers.
2. As firstly done in the presented case, Imatinib medical therapy should be considered to be employed as first line systemic therapy in these patients;
3. Continuing Imatinib medical therapy even after achieving complete radiological response would seem to be a reasonable choice.

## AUTHOR CONTRIBUTIONS

**Simone Rota:** Conceptualization (lead); data curation (lead); project administration (lead); resources (lead); writing – original draft (lead); writing – review and editing (lead). **Andrea Franza:** Data curation (equal); methodology (equal); project administration (equal); writing – original draft (equal). **Chiara Fabbroni:** Conceptualization (equal); investigation (equal); project administration (equal); supervision (equal); validation (equal); visualization (equal). **Biagio Paolini:** Conceptualization (equal); resources (equal). **Francesca Gabriella Greco:** Conceptualization (equal); resources (equal). **Alessandra Alessi:** Conceptualization (equal); resources (equal). **Barbara Padovano:** Conceptualization (equal); resources (equal). **Paolo Casali:** Methodology (lead); supervision (lead); visualization (lead). **Roberta Sanfilippo:** Conceptualization (equal); data curation (equal); formal analysis (equal); investigation (equal); methodology (equal); project administration (lead); resources (equal); supervision (equal).

## FUNDING INFORMATION

This research received no specific grant from any funding agency in the public, commercial, or not‐for‐profit sectors.

## CONFLICT OF INTEREST STATEMENT

Dr Sanfilippo reported receiving personal fees from PharmaMar, Rain Oncology, and Boehringer Ingelheim and fees to her institution from Advenchen Laboratories, Amgen, Bayer, Epizyme, Eli Lilly, Daiichi, GlaxoSmithKline (GSK), Karyopharm, Novartis, Rain Therapeutics, Pfizer, SpringWorks Therapeutics, and PharmaMar outside the submitted work. Dr Fabbroni reported fees to her institution from Advenchen Laboratories, Amgen Dompé, AROG Pharmaceuticals, Bayer, Blueprint Medicines, Deciphera, Epizyme, Eli Lilly, Daiichi Sankyo, GSK, Karyopharm, Novartis, Pfizer, PharmaMar, SpringWorks Therapeutics, and Rain Therapeutics outside the submitted work. Dr Casali reported receiving grants from PharmaMar, Advenchen Laboratories, Amgen Dompé, AROG Pharmaceuticals, Bayer, Blueprint Medicines, Daiichi Sankyo, Eisai, Eli Lilly, Epizyme Inc, GSK, Deciphera, Karyopharm Pharmaceuticals, Novartis, and Pfizer outside the submitted work. The remaining authors declare that the research was conducted in the absence of any commercial or financial relationships that could be construed as a potential conflict of interest.

## ETHICS STATEMENT

Hereby, I, Simone Rota, consciously assure that for the manuscript—*COL1A1::PDGFB* fusion‐associated uterine fibrosarcoma: a case report and review of the literature. The following is fulfilled: This material is the authors' own original work, which has not been previously published elsewhere. The paper is not currently being considered for publication elsewhere. The paper reflects the authors' own research and analysis in a truthful and complete manner. The paper properly credits the meaningful contributions of co‐authors and co‐researchers. The results are appropriately placed in the context of prior and existing research. All sources used are properly disclosed. All authors have been personally and actively involved in substantial work leading to the paper, and will take public responsibility for its content.

## CONSENT STATEMENT

The patient has provided informed consent for the publication of her clinical case details, along with the corresponding images that have been published.

## Data Availability

Data sharing is not applicable to this article as no new data were created or analyzed in this study.
